# Effect of Methanolic Extract of Dandelion Roots on Cancer Cell Lines and AMP-Activated Protein Kinase Pathway

**DOI:** 10.3389/fphar.2017.00875

**Published:** 2017-11-28

**Authors:** Gauhar Rehman, Muhammad Hamayun, Amjad Iqbal, Sumera Afzal Khan, Hamayoon Khan, Adeeb Shehzad, Abdul Latif Khan, Anwar Hussain, Ho-Youn Kim, Jamshaid Ahmad, Ayaz Ahmad, Abid Ali, In-Jung Lee

**Affiliations:** ^1^Department of Zoology, Abdul Wali Khan University Mardan, Mardan, Pakistan; ^2^Department of Botany, Abdul Wali Khan University Mardan, Mardan, Pakistan; ^3^Department of Agriculture, Abdul Wali Khan University Mardan, Mardan, Pakistan; ^4^Centre of Biotechnology and Microbiology, University of Peshawar, Peshawar, Pakistan; ^5^Department of Agronomy, University of Agriculture Peshawar, Peshawar, Pakistan; ^6^Department of Biomedical Engineering and Sciences, SMME, National University of Sciences and Technology (NUST), Islamabad, Pakistan; ^7^UoN Chair of Oman’s Medicinal Plants and Marine Natural Products University of Nizwa, Nizwa, Oman; ^8^Korea Institute of Science and Technology, Gangneung, South Korea; ^9^Department of Biotechnology, Abdul Wali Khan University Mardan, Mardan, Pakistan; ^10^School of Applied Biosciences, Kyungpook National University, Daegu, South Korea; ^11^Research Institute for Dok-do and Ulleung-do Island, Kyungpook National University, Daegu, South Korea

**Keywords:** cancer, AMPK, cytotoxicity, traditional medicine, dandelion

## Abstract

Ethnomedicinal knowledge of plant-derived bioactives could help us in discovering new therapeutic compounds of great potential. Certainly, dandelion has been used in traditional ethno-medicinal systems (i.e., Chinese, Arabian, Indian, and Native American) to treat different types of cancer. Though, dandelion is highly vigorous, but the potential mode of action is still unclear. In the current study, the antiproliferative activity of methanolic extracts of dandelion root (MEDr) on cell viability of HepG2, MCF7, HCT116, and normal Hs27 was investigated. It was observed that MEDr (500 μg/mL) drastically decreased the growth of HepG2 cell line, while the effect on MCF7 and HCT116 cell lines was less pronounced and no effect has been observed in Hs27 cell lines. The MEDr also enhanced the phosphorylation level of AMPK of HepG2 cells, which considered crucial in cancer treatment and other metabolic diseases. The AMPK activation by MEDr noticed in the current study has never been reported previously. The results regarding the number of apoptotic cells (HepG2 cells) were in line with the cell viability test. The current observations clearly demonstrated the potency of MEDr against liver cancer with validation that dandelion could control AMPK and thus cancer in the treated cell lines.

## Introduction

Since ancient time till date, plants have been considered as valuable sources of compounds that can cure various complex diseases, including cancer and diabetes, etc. According to the World Health Organization (WHO), a larger part of the population from the under-developed nations depend on natural resources as a basic medicine. Moreover, 60% of the population around the globe is accustomed to the fruits, vegetables, herbs, and vitamins to prevent and/or treat cancer (Guggenheim and Shah, 2013; Pludowski et al., 2013; Shah et al., 2013; Mishra and Meherotra, 2014).

Dandelion, a common weed that is famous for curing various diseases since primeval times might serve to treat cancer without any side effects (Choi and Kim, 2009; Tettey et al., 2014; Van Trinh et al., 2016). Among the different species of Dandelion, *T. officinale, Taraxacum platycarpum, Taraxacum coreanum*, and *Taraxacum mongolicum* are known for its curative effects (Martinez et al., 2015), but their mode of action is still unclear. Dandelion is famous for its therapeutic property, that is why; it got an important place in traditional medicine system of both native America and China (TCM) to treat heartburn, dyspepsia, anorexia, hepatitis, spleen and liver complaints and cancer (Jeon et al., 2008; Yarnell and Abascal, 2009). The current study was undertaken in this article regarding the effectiveness of MEDr in cancer therapy. Likewise, an attempt was made to demonstrate the effect of various concentrations of the extracts on different cancer lines.

## Materials and Methods

### Reagents

Chemical reagents were of analytical grade and purchased from GIBCO (Burlington, ON, Canada) and Sigma Chemical Co. (St. Louis, MO, United States).

### Cell Cultures

All the cancer cell lines were obtained from United States (American Type Culture Collection). HepG2 and HCT116 cell cultures were nurtured in DMEM and MCF7 cell be cultured in Roswell Park Memorial Institute medium (RPMI). The RPMI medium was supplemented with heat inactivated fetal bovine serum (10%), penicillin (100 unit/ml), and streptomycin (100 μg/ml) at 37°C and 5% CO_2_. Approximately, 1 × 10^6^ cells were cultured on plates at 37°C under a humid environment with 5% CO_2_ for 24 h.

### MTT Assay

The MTT assay, an index of cell viability was carried out by the method as defined by Mosmann (1983).

#### Analysis of Apoptosis by Propidium Iodine (PI) Staining

Briefly, cells were fixed with 70% ethanol at 4°C for overnight. On the next day cells were washed with phosphate-buffered saline (PBS) solution and treated firstly with RNase (0.5 ml) and then with 1 mL of propidium iodide (PI) solution (kept in a dark at 4°C for 30 min before use). After washed, the samples were kept on ice till measured. The DNA histogram was obtained with a flow cytometry cell sorter (Becton Dickinson).

### Methanol Extraction and Sample Fractionation

The dried plant roots were ground in liquid nitrogen and the powdered sample were then extracted with 70% methanol at room temperature for 1 month. The methanol extract (MEDr) evaporated *in vacuo* and was fractioned successively with equal volumes of ethyl acetate (EA) and *n*-butanol (BuOH), leaving a residual aqueous fraction (Aq.). Each fraction was evaporated *in vacuo* to yield EA, BuOH and aqueous residues.

### Protein Extraction and Western Blotting

Cells were initially washed thrice with ice-cold PBS and then lysed in 100–400 μL lysis buffers. After lysis, the cellular debris was removed with the help of centrifuge, operated at 12,000 rpm and 4°C for 20 min. After the quantification of total protein (Bio-Rad, Mississauga, ON, Canada), the clarified protein lysates from each experimental condition was boiled for 5 min. Subsequently, the boiled lysates were subjected to electrophoresis in denaturing 8% SDS-polyacrylamide gel to identify AMPK and acetyl-CoA carboxylase (ACC) proteins. The separated proteins from the SDS-PAGE were then transferred to a nitrocellulose membrane and were blocked. The membranes were later probed with primary antibodies of interest and horseradish peroxidase-conjugated anti-rabbit IgG as secondary antibody. Finally, the position of proteins was visualized using the enhanced chemiluminescene (ECL) reagent.

### Statistical Analysis

All the experiments were replicated and the data presented as mean ± SE. The significant differences between the means were computed by *t*-test using Microsoft Excel, 2010.

## Results

### Effect of MEDr on Cell Viability

**FIGURE 1:**
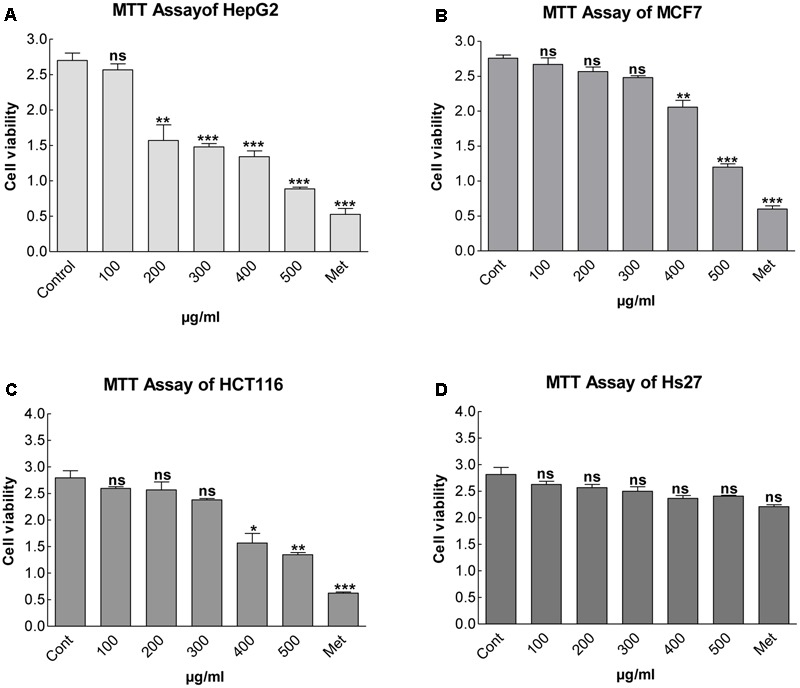
Cell viability as influenced by various concentrations of methanolic extracts of dandelion root (MEDr). The cells (5 × 10^5^ cells/well) were incubated for 24 h with various concentrations of MEDr. The abbreviation Cont. represent control, i.e., the cells were incubated without MEDr; Met represents Metformin. The cell viability was assessed by MTT assay. Each data point indicates the mean of three separated experiments with ± SE. **(A)** Represents MTT assay for HepG2 cell line; ns = non-significant at *P* = 0.01; Significant differences from the control are indicated: ^∗∗^*P* < 0.01; ^∗∗∗^*P* < 0.001. **(B)** Represents MTT assay for MCF7 cell line; ns = non-significant at *P* = 0.01; Significant differences from the control are indicated: ^∗∗^*P* < 0.01; ^∗∗∗^*P* < 0.001. **(C)** Represents MTT assay for HCT116 cell line; ns = non-significant at *P* = 0.01; Significant differences from the control are indicated: ^∗^*P* < 0.01; ^∗∗^*P* < 0.001, ^∗∗∗^*P* < 0.0001. **(D)** Represents MTT assay for Hs27 (human foreskin fibroblast cell line); ns = non-significant at *P* = 0.01.

The effect of MEDr on the cell viability of HepG2, MCF7, and HCT116 cells assessed by the MTT test (**Figures 1A–C**). The cells (5 × 10^3^ cells/well) incubated in a culture medium having various concentrations (0, 100, 200, 300, 400, 500 μg/mL) of MEDr for 24 h. MEDr at a concentration of 200 μg/ml reduced the cell viability by 62%, whereas at 500 μg/ml, the cell viability reduced to almost 20% with respect to the control treatment. The result clearly demonstrated that MEDr caused a significant dose-dependent reduction in cell viability of HepG2 cells, while its effect on MCF7 and HCT116 cell lines were comparatively low. On the other hand, MEDr didn’t show any effect on the viability of normal Hs27, human foreskin fibroblast cell line (**Figure 1D**).

### Dose-Dependent AMPK Activation by MEDr

**FIGURE 2:**
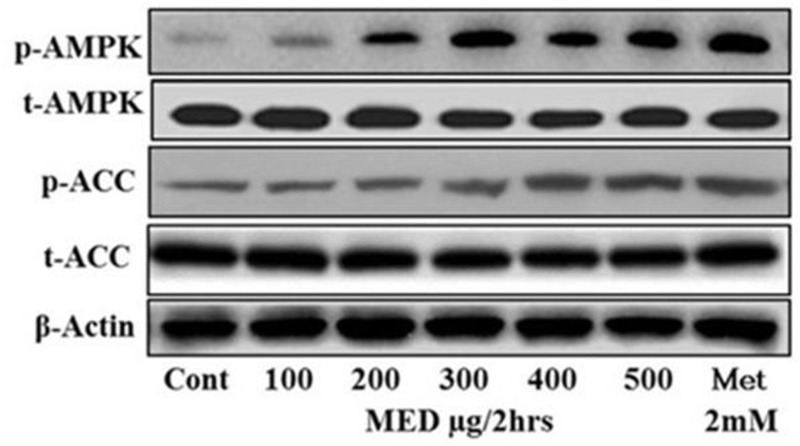
Dose-dependent effect of MEDr on AMPK activation. HepG2 cells were incubated with the indicated concentrations of MEDr for 2 h. The phosphorylation of AMPK and ACC as a readout for the activity of AMPK was analyzed through Western blot.

The effect of MEDr on the activation of AMPK in HepG2 cells was analyzed by western blot (**Figure 2**). The HepG2 cells seeded in 100 mm dish and on 80–90% confluence; the cells were then treated with the various concentrations (0, 100, 200, 300, 400, 500 μg/ml) of MEDr. The western blot data showed that the MEDr activated AMPK in a dose-dependent manner. At a concentration of 200 μg/ml, MEDr activated the AMPK, however, maximum activity of AMPK achieved at a concentration of 500 μg/ml. Similarly, the phosphorylation level of ACC also increased with an increase in the concentration of MEDr.

### Time-Dependent AMPK Activation by MEDr

**FIGURE 3:**
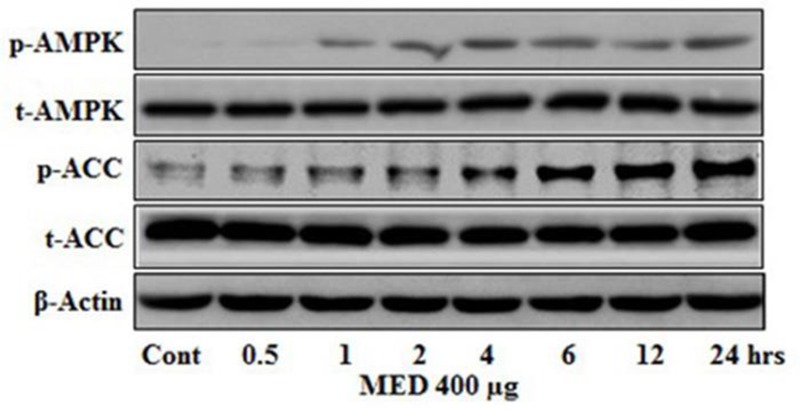
Time-dependent effect of MEDr on AMPK activation. HepG2 cells were incubated with 400 μg/ml of MEDr from 30 min to 24 h. The phosphorylation of AMPK and ACC as a readout for the activity of AMPK was analyzed by Western blot.

Time-dependent effect of MEDr on the activation of AMPK in HepG2 cells is presented in **Figure 3**. The HepG2 cells were seeded in 100 mm dish and on 70–80% confluence, the cells were then treated with the 400 μg/ml concentration of MEDr for various time points (0.5, 1, 2, 4, 6, 12, and 24 h). In the time-dependent experiment, the AMPK activation initiated at the first hour that increased gradually with time and reached to a peak value at 24 h. The phosphorylation level of ACC also increased in a similar fashion to that of AMPK.

### Influence of MEDr on Cell Apoptosis

**FIGURE 4:**
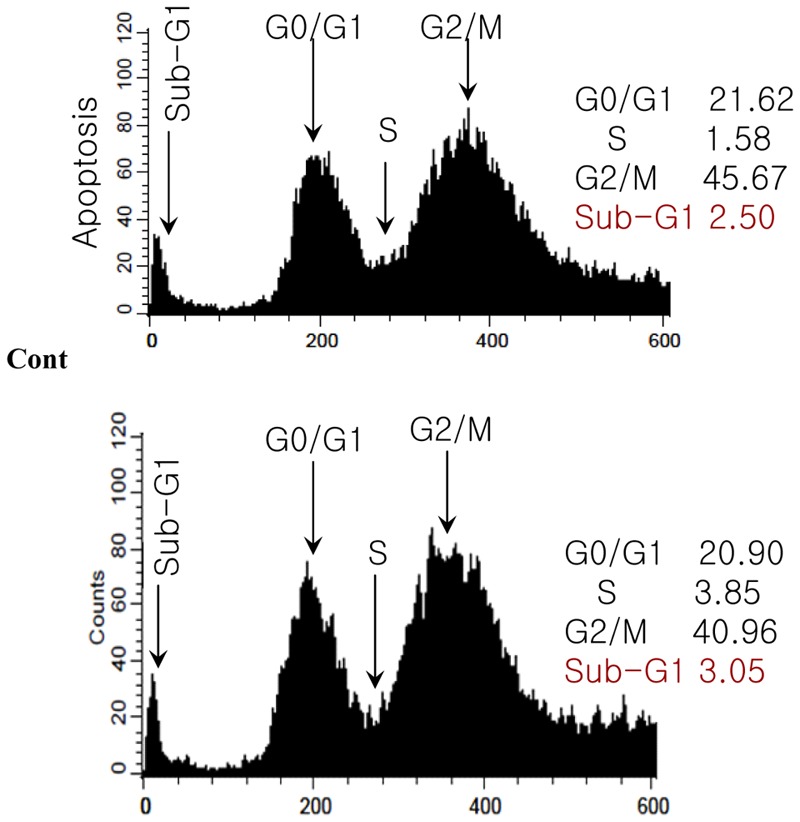
Cell cycle distribution of HepG2 cells as influenced by MEDr. The cells were incubated for 24 h in medium alone (control) or with MEDr (500 μg/ml). The cells were stained with PI solution and analyzed for DNA content by flow cytometery.

HepG2 cancer cells were exposed to various concentrations of MEDr and the apoptosis was analyzed by flow cytometery assay. The cell cycle distribution of cells treated with MEDr was observed after 24 h of incubation (**Figure 4**). Though the effect was mild, but the result showed that the cell cycle was arrested at the sub G1 phase. Such observation indicated that apoptosis was the main cause that inhibited the cell proliferation. The pattern of apoptosis was consistent to that of cell viability test.

**FIGURE 5:**
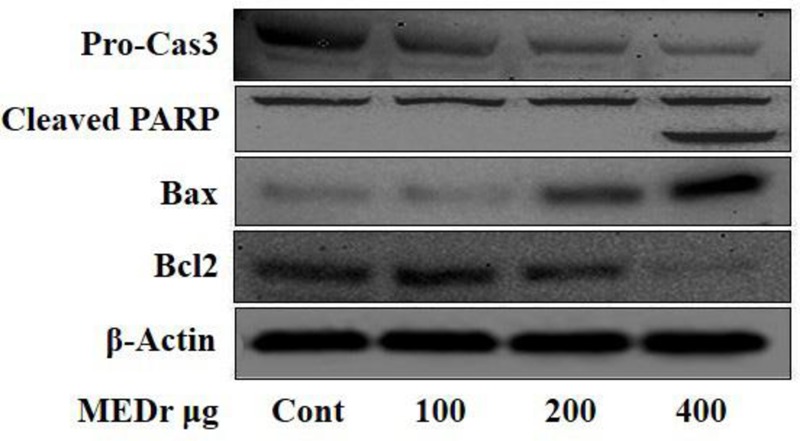
Dose dependent effect of MEDr on apoptotic markers.

To confirm it further, HepG2 cells were treated with MEDr in a dose dependent manner for 2 hrs. The results of Western blot exhibited that MEDr are capable of down-regulating antiapoptotic factor (Bcl-2) and up-regulating the pro-apoptotic factor (Bax), thereby decreasing the Bcl-2/Bax ratio and exposing cells to apoptosis (**Figure 5**). Interestingly, MEDr induced apoptosis with an increased serine phosphorylation level of AMPK, which trans-activates Bax expression. Similarly, a reduction in pro-caspase-3 was seen in response to MEDr treatment. In addition, the PARP-1 cleavage fragment was observed in cells following exposure to MEDr. These data indicate that MEDr exerts much of its suppressive effect on cancer cells by the induction of apoptosis (**Figure 5**).

### Prediction of Possible Bioactive Compounds in Dandelion Extract Through Docking

**FIGURE 6:**
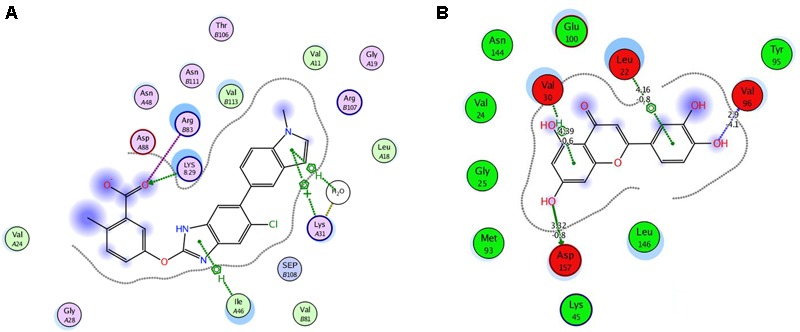
Interaction of selected ligands **(A)** reference ligand 991 and **(B)** test ligand luteolin-4-0 glucoside with the kinase domain of AMPK protein.

The phytochemicals found in Dandelion (Katrin et al., 2006; Bing et al., 2013) plant were docked with the energy minimized structure of AMPK, PDB accession 4CFE. Luteolin 7-*O*-glucoside and Luteolin 7-glucoside showed a highest degree of interaction with the kinase domain of AMPK as shown by a docking score of –17.4 and –17.3, respectively. However, their site of interaction was different from the known activator, the reference compound 991. Interestingly, Luteolin 4-*O*-glucoside docked with relatively low energy (–9.5) but shared the same pocket with the standard AMPK activator 991 (**Figure 6**). Nevertheless, the later ligands interacted with different amino acids.

## Discussion

Handful of synthetic drugs has been developed and tested clinically against cancer, however, these drugs have shown side effects during the pre- and pro-clinical trials. Herbal drugs are, therefore, got attention during recent times to cure cancer and other prevalent diseases. Plants belonging to the genus *Taraxacum* in general and dandelion, in particular have proved to be placed in traditional medicine to alleviate various diseases, including cancer. However, the information regarding the anticancer activity of dandelion is anecdotal. Also, very little is known till date, concerning the therapeutic effect of dandelion in cancer. In the present study, we have clearly demonstrated that the growth of cancer cells was significantly inhibited by dandelion in a dose-dependent manner. Likewise, the cytotoxic effect of the MEDr was suggested by the MTT assay and was translated by the activation of AMPK (a sensor of cellular energy). Previously, dandelion extracts were reported to contain triterpenoids, sesquiterpenes, and phenolic compounds that played vital role in inhibiting tumor cell proliferation (Murtaza et al., 2009; Lefanc et al., 2017). Since, the roots of dandelion are abundant in these compounds; it might be possible that one of these compounds has an inhibitory effect. Our results are in line with those of Lambert et al. (2005), who described an inhibitory effect of these compounds, particularly the effect of polyphenols on cancer cell proliferation.

Earlier, the extracts obtained from different parts (i.e., leaves, flowers, and root) of dandelion have proved to be active against cancer by affecting the extracellular signal-regulated kinase (ERK) pathway (Sigstedt et al., 2008). Since, there is a link between ERK and AMPK activation; it is possible that MEDr inhibited cancer by activating AMPK pathway as shown in HepG2 cell line. The activation of AMPK was caused by the application of MEDr in a dose-dependent manner. Other evidence that revealed the effectiveness of MEDr in cancer therapy comes from the results of apoptosis experiment. This evidence is further supported by the results of this study, where MEDr has an effect on apoptotic factors, i.e., Bcl-2, Bax, and PARP-1. Bcl-2 family proteins c as regulators of apoptosis and the Bcl-2 family can act as checkpoints, which determine the cell death or survival through signals before cell enters into apoptosis (Cory and Adams, 2002). Another study shows that metformin activates AMPK and inactivates Akt (an upstream regulator of mTOR). Simultaneously, metformin inactivates p70S6K, a downstream player of this pathway, backup the argument that AMPK/p70S6K pathway is a major target of metformin to induced apoptosis in MCF-7 cells (Queiroz et al., 2014). Parallel to this study there are evidences that by direct phosphorylation, Bax can be inhibit by activation of AKT in human lung cancer cells (Xin and Deng, 2005). Furthermore, the activation of the AMPK-mediated apoptotic signaling pathway may play an important role in apoptosis by modulating the Bcl-2/Bax ratio, as also reported for breast cancer cells (Choudhuri et al., 2002). Likewise, PARP-1 cleavage and a reduction in the level of pro-caspase-3 due to caspase activity are indicators of apoptosis (Wolf and Green, 1999). In short, apoptosis is one of the forms of programmed cell death (PCD) that plays an important role in different cellular processes (such as, growth regulation, neurodegenerative disorders, heart diseases and cancer) (Gibson, 2001; Parton et al., 2001). In our case, MEDr significantly induced cell death in HepG2 cancer cell line, suggesting that dandelion can be used to inhibit the growth of tumor cells.

## Conclusion

From the results of the current study it was concluded that MEDr inhibited the cancer cell proliferation by activating AMPK. It also authenticated the potential and efficient use of natural bioactive compounds in our primary health care. The compounds from natural sources, such as dandelion can serve as a reservoir of potent bioactive compounds that might inhibit different form of cancer without any side effects. Furthermore, the rigorous fractionation and isolation of the active principle(s) present in the various extracts of dandelion would play a crucial role in explaining the anti-proliferative effect of dandelion on HepG2, MCF7 and HCT116 cancer cells.

## Author Contributions

GR, MH, AI, and SAK designed the project. AS, ALK, HK, HYK, JA, AyA, and AbA performed the experiments. GR, AI, and AH analyzed the data. IL and MH supervised and funded the project. GR and AI wrote the draft manuscript.

## Conflict of Interest Statement

The authors declare that the research was conducted in the absence of any commercial or financial relationships that could be construed as a potential conflict of interest.

## References

[B1] Bing X. Matthew J. S. David C. Nicola J. B. Lesley F. H. Elizabeth U. (2013). Structural basis of AMPK regulation by small molecule activators. *Nat. Commun.* 4:3017. 10.1038/ncomms4017 24352254 PMC3905731

[B2] Choi E. J. Kim G.-H. (2009). Dandelion (*Taraxacum officinale*) flower ethanol extract inhibits cell proliferation and induces apoptosis in human ovarian cancer SK-OV-3 cells. *Food Sci. Biotechnol.* 18 552–555.

[B3] Choudhuri T. Pal S. Agwarwal M. L. Das T. Sa G. (2002). Curcumin induces apoptosis in human breast cancer cells through p53-dependent Bax induction. *FEBS Lett.* 512 334–340. 10.1016/S0014-5793(02)02292-5 11852106

[B4] Cory S. Adams J. M. (2002). The Bcl2 family: regulators of the cellular life-or-death switch. *Nat. Rev. Cancer* 2 647–656. 10.1038/nrc883 12209154

[B5] Gibson R. M. (2001). Does apoptosis have a role in neurodegeneration? *Br. Med. J.* 322 1539–1540. 10.1136/bmj.322.7301.1539 11420281 PMC1120578

[B6] Guggenheim D. E. Shah M. A. (2013). Gastric cancer epidemiology and risk factors. *J. Surg. Oncol.* 107 230–236. 10.1002/jso.23262 23129495

[B7] Jeon H.-J. Kang H.-J. Jung H.-J. Kang Y.-S. Lim C.-J. Kim Y.-M. (2008). Anti-inflammatory activity of *Taraxacum officinale*. *J. Ethnopharmacol.* 115 82–88. 10.1016/j.jep.2007.09.006 17949929

[B8] Katrin S. Reinhold C. Andreas S. (2006). Taraxacum—a review on its phytochemical and pharmacological profile. *J. Ethnopharmacol.* 107 313–323. 10.1016/j.jep.2006.07.021 16950583

[B9] Lambert J. D. Hong J. Yang G.-Y. Liao J. Yang C. S. (2005). Inhibition of carcinogenesis by polyphenols: evidence from laboratory investigations. *Am. J. Clin. Nutr.* 81 284S–291S. 15640492 10.1093/ajcn/81.1.284S

[B10] Lefanc F. Tabanca N. Kiss R. (2017). Assessing the anticancer effects associated with food products and/or nutraceuticals using in vitro and in vivo preclinical development-related pharmacological tests. *Semin. Cancer Biol.* 46 14–32. 10.1016/j.semcancer.2017.06.004 28602819

[B11] Martinez M. Poirrier P. Chamy R. Prüfer D. Schulze-Gronover C. Jorquera L. (2015). *Taraxacum officinale* and related species—an ethnopharmacological review and its potential as a commercial medicinal plant. *J. Ethnopharmacol.* 169 244–262. 10.1016/j.jep.2015.03.067 25858507

[B12] Mishra A. Meherotra R. (2014). Head and neck cancer: global burden and regional trends in India. *Asian Pac. J. Cancer Prev.* 15 537–550. 10.7314/APJCP.2014.15.2.537 24568456

[B13] Mosmann T. (1983). Rapid colorimetric assay for cellular growth and survival: application to proliferation and cytotoxicity assays. *J. Immunol. Methods* 65 55–63. 10.1016/0022-1759(83)90303-4 6606682

[B14] Murtaza I. Saleem M. Adhami V. M. Hafeez B. B. Mukhtar H. (2009). Suppression of cFLIP by lupeol, a dietary triterpene, is sufficient to overcome resistance to TRAIL-mediated apoptosis in chemoresistant human pancreatic cancer cells. *Cancer Res.* 69 1156–1165. 10.1158/0008-5472.CAN-08-2917 19176377 PMC2996261

[B15] Parton M. Dowsett M. Smith I. (2001). Studies of apoptosis in breast cancer. *Br. Med. J.* 322 1528–1532. 10.1136/bmj.322.7301.1528 11420276 PMC1120573

[B16] Pludowski P. Holick M. F. Pilz S. Wagner C. L. Hollis B. W. Grant W. B. (2013). Vitamin D effects on musculoskeletal health, immunity, autoimmunity, cardiovascular disease, cancer, fertility, pregnancy, dementia and mortality—a review of recent evidence. *Autoimmun. Rev.* 12 976–989. 10.1016/j.autrev.2013.02.004 23542507

[B17] Queiroz E. A. Puukila S. Eichler R. Sampaio S. C. Forsyth H. L. Lees S. J. (2014). Metformin induces apoptosis and cell cycle arrest mediated by oxidative stress, AMPK and FOXO3a in MCF-7 breast cancer cells. *PLOS ONE* 9:e98207. 10.1371/journal.pone.0098207 24858012 PMC4032293

[B18] Shah U. Shah R. Acharya S. Acharya N. (2013). Novel anticancer agents from plant sources. *Chin. J. Nat. Med.* 11 16–23. 10.3724/SP.J.1009.2013.00016

[B19] Sigstedt S. C. Hooten C. J. Callewaert M. C. Jenkins A. R. Romero A. E. Pullin M. J. (2008). Evaluation of aqueous extracts of *Taraxacum officinale* on growth and invasion of breast and prostate cancer cells. *Int. J. Oncol.* 32 1085–1090. 10.3892/ijo.32.5.1085 18425335

[B20] Tettey C. Ocloo A. Nagajyothi P. Lee K. (2014). An *in vitro* analysis of antiproliferative and antimicrobial activities of solvent fractions of *Taraxacum officinale* (Dandelion) leaf. *J. Appl. Pharm. Sci.* 4 41–45.

[B21] Van Trinh N. Dang N. D.-P. Tran D. H. Van Pham P. (2016). *Taraxacum officinale* dandelion extracts efficiently inhibited the breast cancer stem cell proliferation. *Biomed. Res. Ther.* 3 733–741. 10.7603/s40730-016-0034-4

[B22] Wolf B. B. Green D. R. (1999). Suicidal tendencies: apoptotic cell death by caspase family proteinases. *J. Biol. Chem.* 274 20049–20052. 10.1074/jbc.274.29.20049 10400609

[B23] Xin M. Deng X. (2005). Nicotine inactivation of the proapoptotic function of Bax through phosphorylation. *J. Biol. Chem.* 280 10781–10789. 10.1074/jbc.M500084200 15642728

[B24] Yarnell E. Abascal K. (2009). Dandelion (*Taraxacum officinale* and *T. mongolicum*). *Integr. Med.* 8 35–38.

